# Leveraging Random Forests explainability for predictive modeling of children's conduct problems: insights from individual and family factors

**DOI:** 10.3389/fpubh.2025.1526413

**Published:** 2025-06-12

**Authors:** Estrella Romero, Jaime González-González, María Álvarez-Voces, Enrique Costa-Montenegro, Beatriz Díaz-Vázquez, Andrea Busto-Castiñeira, Paula Villar, Laura López-Romero

**Affiliations:** ^1^Department of Clinical Psychology and Psychobiology, Institute of Psychology (IPsiUS), University of Santiago de Compostela, Campus Vida, Santiago de Compostela, Spain; ^2^atlanTTic, Information Technologies Group, Universidade de Vigo, Vigo, Spain

**Keywords:** conduct problems, childhood, Random Forest, family variables, individual variables, explainability

## Abstract

Conduct problems are among the most complex, impairing, and prevalent challenges affecting the mental health of children and adolescents. Due to their multifaceted nature, it is important to develop predictive models that capture the intricate interactions among contributing factors. This longitudinal study aims to: (1) evaluate the utility and effectiveness of Random Forest models for classifying children with varying levels of conduct problems, (2) analyze the interactions between individual and family variables in predicting high levels of conduct problems, and (3) determine the most relevant factors or combinations for accurate child classification. The sample was drawn from the ELISA study, and consisted of 1,352 children assessed twice within a 1-year frame. The use of Random Forest and its inherent structure allowed to identify subsets of variables with the capability of predicting Conduct Problems in children. This research demonstrates the effectiveness of integrating psychological insights with advanced computational techniques to address critical concerns in children's mental health, emphasizing the need for enhanced screening and tailored interventions.

## 1 Introduction

Child conduct problems (CP) comprise different forms of behavioral maladjustment, including aggressive, oppositional, defiant, deceitful, and rule-breaking behavior that violates the rights of others and conflicts with societal norms (1, 2). Both at clinical and subclinical levels, CP represents one of the most relevant, impairing, and prevalent problems across childhood and adolescence, being the primary reason for referring children to psychoeducational and mental health services (3, 4).

Epidemiologic studies have shown prevalence rates of CP in community samples ranging from 2% to more than 10% (2), around 8%–10% engage in the more severe childhood-onset form of CP, and another 25% initiate clinically significant levels of CP during adolescence (5). The severity of CP is endorsed by the wide range of deleterious outcomes linked to early manifestations of CP, which include a host of social, emotional, and academic problems such as anxiety and depression, substance use and abuse, academic failure and underachievement, impaired family well-being, problems in peers and intimate relationships, and legal system involvement (5–7).

Of note, a recent study conferred unique importance to CP as the leading cause of burden among all mental disorders identified up to age 14 (8). However, CP consequences are not only limited to the individual or family levels, with long-term societal [e.g., higher levels of service usage (9)] and economic burden (10) that place CP as an issue of public concern.

### 1.1 Understanding child CP: disentangling involved factors

Considering the wide range of personal and social implications related to CP, many efforts have been made to better understand the causes behind this phenomenon and to identify all the factors involved in its development and stability over time. Research conducted in this field has resulted in a long list of dispositional and contextual indicators that may impact the development of CP.

At the child (individual) level, developmental models have listed, based on previous research, some temperamental, personality, and socio-emotional variables with proven influence in the emergence of CP. Among the temperamental variables, widely analyzed in the context of child CP [e.g., (11)], two facets of temperament have demonstrated strong prediction for later maladjustment (12). When present at adaptive levels, emotion regulation, which refers to the ability to regulate emotional experience and expression in favor of adaptive behavior (13), and effortful control, defined as the self-regulatory aspect of temperament that involves attentional and inhibitory control mechanisms (14, 15), have evidenced their role as protective factors that may buffer the development of child CP [e.g., (16)]. Conversely, emotional and behavioral dysregulation would stem from emotional lability, negative affect, and poor effortful control, linked with an increased risk for later CP (12, 17).

Beyond the temperamental influence on child CP, personality traits also play an essential role in the early manifestation and later development of CP. In recent years, there has been an increasing interest in examining traits encompassing interpersonal [e.g., grandiose-deceitful (GD)], affective [e.g., callous-unemotional (CU)], and behavioral features [e.g., impulsive-need for stimulation (INS)] that resemble adult psychopathic personality (18).

Psychopathic traits in childhood have proven their usefulness and predictive value in the prediction of more severe and persistent pathways of CP (19, 20), even when controlling for other relevant risk factors [e.g., (21)]. This influence is particularly noteworthy when all psychopathic dimensions are present, reinforcing the importance of interpersonal (GD) and behavioral (INS) traits beyond the well-known influence of CU traits on child CP (22, 23).

Related to the aforementioned temperamental and personality variables, socioemotional competence has shown a particular ability to promote or restrain child CP based on their adaptive or maladaptive functioning. Deficits in emotion regulation linked to CU traits may drive deficits in the ability to understand and resonate with other's emotions. Empathy has been defined as a multi-component process involving at least affective (i.e., automatic emotional reaction to someone else's emotions) and cognitive domains (i.e., ability to recognize, understand, and share other people's emotions and perspectives), both of them related with multiple forms of CP in childhood and adolescence (24, 25).

Beyond the individual factors, the complexity underlying the CP should be interpreted in the context of the dynamic interplay between the individual and environmental domains. In this regard, individual vulnerabilities unfold in relational contexts, where the child interacts with peers, adults, and systems around them (26).

At early developmental stages, the family constitutes the most proximal context for child development, building social and emotional skills that will support later adjustment (27). Therefore, it is unsurprising that a large set of family variables, with the ability to promote or restrain child adjustment, were repeatedly placed in developmental models of CP (28–30).

From the family context, variables focused on the parent-child relationship have received much attention. The influence of parenting practices, including both positive (e.g., parental responsiveness and warmth) and ineffective parenting (e.g., permissiveness, harsh parenting), in the development of child CP has been extensively recognized in previous research (31). Ineffective and negative parenting, which a difficult child temperament might fuel, is expected to lead to tensions between parents and children, increasing conflictive interactions in the dyad that, in turn, may place the child at risk for CP [e.g., (32)].

Instability in parent-child relationships could also increase the risk for parenting stress, also triggered by an overwhelmed feeling from the multiple challenges and demands required in parenting, which were already related to the emergence and maintenance of CP (33, 34). Additional sources of parental stress (e.g., financial problems, job demands, mental health issues) have also been recognized as potential risk factors for child CP [e.g., (35, 36)]. In this regard, different forms of non-severe psychopathology, including perceived stress, anxiety, and depressive symptoms, have also shown their influence on the development of CP, being considered one of the most relevant early predictors of persistence in the most severe forms of later problematic behavior (37).

### 1.2 Integrating individual and family factors in the prediction of CP: a machine learning approach

Understanding child CP in-depth implies considering its complexity, largely represented by the large set of individual and environmental factors spanned in previous research. As occurs with other mental health problems, most of these factors repeatedly emerged in different studies investigating a reduced number of factors or domains at a time, resulting in a vast array of predictors of CP collected in previous literature but with limited information about their relative importance in prediction (38). Also, predictive models of CP have benefited when a cumulative risk perspective was assumed instead of focusing on one or two risk factors [e.g., (39)], suggesting that more complex and varied approaches, allowing to handle a large number of predictors, are needed to improve classification and prediction of child CP.

However, dealing with the multi-factor nature of CP through traditional data-driven approaches (e.g., regression models) has come with several limitations related to mass univariate testing, non-linearity, interaction effects, or overfitting, among others (40, 41), raising the need for new advanced and more sophisticated methodological procedures.

### 1.3 Why machine learning in longitudinal data

We face several challenges in trying to predict some health topics based on multiple features. As previously mentioned, these aspects of prediction have been traditionally addressed by methods such as regression. In this sense, Machine Learning (ML), still in its infancy in longitudinal data, can provide some valuable advantages (42).

Firstly, incorporating numerous repeated measurements of exposures presents significant challenges because traditional regression models are typically not designed to handle many covariates (43). Secondly, these regression models often assume a linear relationship between each exposure and the outcome, with few or no specified interactions between exposures. However, these assumptions are often unverifiable; if they are incorrect, they can lead to erroneous conclusions. Despite this, these assumptions are frequently overlooked or violated, which can bias the study results (44, 45).

In this sense, ML provides a solution to these limitations of traditional statistical methods because ML can process vast amounts of data with numerous exposures, automatically developing models that accurately predict outcomes and identify the most critical predictive exposures (46, 47). Importantly, ML techniques generally do not assume a specific functional form for the model; instead, they attempt to derive the model directly from the data to maximize prediction accuracy (48).

### 1.4 Related works

ML approaches allow for classification and prediction in a multivariate context, projecting data into a multi-dimensional nature space where a classifier can create a decision boundary that optimally separates individuals of different classes within this space, whilst all the factors and their interactions are simultaneously considered (49).

Random Forest (RF), a ML algorithm based on Decision Trees (DT), works under these premises, allowing classification and prediction by handling a large number of predictors that can be distilled based on their relative importance. RF models generate multiple DTs to produce aggregated predictions, detecting interactions and non-linear patterns and reducing overfitting, which overcomes most of the limitations observed in traditional regression models (41).

RF has been increasingly used in the prediction of different mental health problems [e.g., (38, 50)], although its use for predicting and classifying child CP is, to date, limited. Alternative ML classifiers have been used to classify CP children based on parenting practices (51) and emotion recognition abilities (49). At the same time, only one study has included multiple risk factors from multiple domains (i.e., biological, psychological, and social) in predicting child CP over a 2-year period (52). Remarkably, none of the previous studies have addressed the classification of individuals according to levels of CP.

The literature on the application of ML in psychology and healthcare often addresses the idea of explainability (53, 54). According to DARPA's Explainable Artificial Intelligence (XAI) program, explainability refers to an artificial intelligence (AI) system's ability to articulate its reasoning comprehensibly to human users. In recent years, XAI gained relevance within the domains of psychology and psychiatric diagnosis, where decision-making should always be supervised by specialists (55).

In contrast to opaque ML systems, the transparency XAI offers enables health experts to verify AI-assisted classifications, ensuring they are fair, unbiased, and ethically sound. This mitigates misclassification by revealing the most influential factors contributing to a potential diagnosis. Notable applications of explainability and ML include early detection of ADHD (56), schizophrenia diagnosis (57), prediction of anxiety and depression (58, 59), and Alzheimer's disease classification (60). However, to our knowledge, this is the first study addressing the detection of CP using ML with explainability techniques.

### 1.5 The current study

Given the multifaceted nature of CP in children, it is essential to explore predictive models that can account for the intricate interplay of its contributing variables. This study leverages a data-driven approach and, based on longitudinal data collected with a 1-year interval, conducts a ML approach for classifying children according to their levels of CP, considering both individual and family predictors.

This study is based on RF, a supervised ML method that combines the results of many different DTs. The longitudinal perspective enables the use of supervised methods, making possible the prediction of future CP using current information about individual and family factors. This also helps improve prediction accuracy by identifying interactions and patterns that traditional statistical models, like linear relationships, cannot capture.

Specifically, this study aims to focus on the following objectives: (1) To examine and describe the utility and performance of RF models for classifying children with different levels of CP based on individual and family factors; (2) To analyze the interplay of individual and family variables for predicting high CP; (3) To determine which factors, or combination of them, are more relevant for child classification, especially to classify children high on CP; (4) To compare decision-making models based on the presence or absence of a prior measure of CP, analyzing the predictive power of other variables when no prior CP are present.

## 2 Materials and methods

### 2.1 Participants

The sample consisted of 1,352 children (51.2% girls) from the longitudinal ELISA project, which is carried out in Galicia (northwestern Spain). For this study, two waves of the project were utilized: T1 (2022; M_age_ = 9.20, SD = 1.04, age range = 7–11 years) and T2 (2023; M_age_ = 10.24, SD = 1.05, age range = 8–12 years). Most of the children were Spanish (~95%).

Regarding the parents' level of education, 11.8% of the mothers and 25.4% of the fathers had attained the highest level of compulsory school education. Additionally, 8.4 and 10% of the mothers and fathers, respectively, had completed a higher level of non-compulsory education. A total of 26.3 and 29.9% had completed vocational training studies, 44.4 and 27.7% had completed university studies, and 8.9 and 6% had completed postgraduate studies. In T1 (2022), 87.1% of mothers and 92.7% of fathers worked outside the home, 5.3 and 2.7% were unemployed or on temporary layoff, 1 and 3.7% were retired/disabled, or unable to work, 0.9 and 0.4% were students, and 5.7 and 0.7% were responsible for domestic duties. In terms of financial well-being, 56.3% of families reported being financially comfortable, 37.4% reported barely getting by, and 6.4% reported having difficulty or serious problems making ends meet. Regarding concern over financial obligations, 39.3% of families reported never worrying, 25.8% worried less than once a month, 31.4% worried at least monthly, and 3.5% worried almost every day.

### 2.2 Measures

#### 2.2.1 Children's predictors (T1)

##### 2.2.1.1 Children's temperament variables

Attention focusing and inhibitory control were measured with the Temperament in Middle Childhood Scale (TMCQ) subscales of the same name (61). The attention-focusing subscale comprised seven items (e.g., “Has a hard time paying attention”; α = 0.92), and inhibitory control comprised eight items (e.g., “Can stop him/herself from doing things too quickly”; α = 0.73). The response scale of the questionnaire is a 5-point Likert-type scale, from 1 (totally false) to 5 (totally true).

##### 2.2.1.2 Children's psychopathic traits

Children's psychopathic traits were examined using the Child Problematic Traits Inventory (CPTI) (18). This instrument consists of 28 items with a 4-point Likert response scale ranging from 1 (does not apply very well) to 4 (applies very well). Eight items are used to measure GD (e.g., “Lies often to avoid problems”; α = 0.84), 10 to measure CU (e.g., “Does not become upset when others are being hurt”; α = 0.88) and 10 to measure INS (e.g., “Often has difficulties with awaiting his or her turn”; α = 0.86).

##### 2.2.1.3 Socioemotional competence

Emotional regulation and lability/negativity were measured through the Emotion Regulation Checklist (ERC) (62). This scale consists of 24 items with a 4-point Likert-type response scale ranging from 1 (never) to 4 (almost always). The emotional regulation subscale consists of eight items (e.g., “Responds positively to neutral or friendly overtures by adults”; α = 0.70) and the lability/negativity subscale of 16 items [e.g., “Is impulsive (cannot control him/herself)”; α = 0.83].

Children's cognitive and affective empathy were measured by items based on Griffith's scale (63). Both variables were measured by three items: cognitive empathy (e.g., “Does not seem to understand why people get upset”; α = 0.83) and affective empathy (e.g., “Gets sad when he sees movies or something sad on TV”; α = 0.75). The response scale was a 4-point Likert-type scale ranging from 0 (strongly disagree) to 3 (strongly agree).

##### 2.2.1.4 Children's CP

The Conduct Problems Scale (18) was used to measure CP in children. This questionnaire consists of 10 items (e.g., “Threatens others”; α = 0.87) with a 5-point Likert-type response scale ranging from 1 (never) to 5 (almost always).

#### 2.2.2 Parenting predictors (T1)

##### 2.2.2.1 Dysfunctional parenting practices

The Parenting Scale Short Form (PS-8) (64) was employed to assess parental overreactivity and laxness. This questionnaire comprises eight items and two subscales, each comprising four items. The first subscale measures overreactivity (e.g., “When my child misbehaves, I raise my voice or shout”; α = 0.74), while the second subscale concerns laxness (e.g., “I am the kind of mother/father who lets her child do what they want”; α = 0.75). The response scale of the questionnaire is a 5-point Likert-type scale ranging from 1 (never) to 5 (always).

##### 2.2.2.2 Child-parent conflict

The Child-Parent Relationship Scale-Short Form [CPRS-SF; (65)] was employed to assess child-parent conflict. The scale comprises eight items (e.g., “My child easily becomes angry at me”; α = 0.85), with a 5-point Likert-type response scale from 1 (definitely does not apply) to 5 (definitely applies).

##### 2.2.2.3 Parental warmth

Parental warmth was measured by six items based on the Child Rearing Scale (66). The items (e.g., “We shared pleasant and loving moments together”; α = 0.84) had a 5-point Likert-type response scale ranging from 1 (never) to 5 (always).

##### 2.2.2.4 Parenting stress

Parenting stress was measured using a scale based on the Parental Stress Scale (PSS) (67). This scale consisted of five items (e.g., “I feel overwhelmed by the responsibilities of being a parent”; α = 0.73) with a 5-point Likert-type response scale ranging from 1 (strongly disagree) to 5 (strongly agree).

#### 2.2.3 Parents characteristics (T1)

##### 2.2.3.1 Parental anxiety and depressive symptoms

The Patient Health Questionnaire-4 (PHQ-4) (68) was used to measure the presence of anxiety and depressive symptoms. This brief scale consists of four items from two different subscales with two items each: anxiety (e.g., “Feeling nervous, anxious or on edge”; α = 0.84) and depression (e.g., “Feeling down, depressed or hopeless”; α = 0.81). The response scale is a 4-point Likert scale ranging from 0 (not at all) to 3 (almost every day).

##### 2.2.3.2 Parents' perceived general stress

Perceived stress was measured using the Perceived Stress Scale-Short Form (PSS-4) (69). This four-item scale (e.g., “In the last month, how often have you felt that you were unable to control the important things in your life?”; α = 0.75) has a 5-point Likert-type response scale ranging from 0 (never) to 4 (very often).

##### 2.2.3.3 Parents' perceived support

Emotional support and instrumental support received were measured through the subscales of the BRIEF-2 Social Support Scale (70). Both subscales were measured through three items each: emotional support received (e.g., “When I am feeling down, there is someone I can lean on”; α = 0.95) and instrumental support received (e.g., “There is someone who can help me fulfill my responsibilities when I am unable to”; α = 0.89). The response scale of the questionnaire was a 6-point Likert-type scale ranging from 0 (never) to 5 (always).

#### 2.2.4 Outcome (T2)

##### 2.2.4.1 Children's CP

Children's CP were measured through the Conduct Problem Scale (18), see Section 2.2.1 for more information. The children were divided into three groups based on their score on the Conduct Problem Scale at T2. The classification was as follows: children scoring below or equal to –0.5 SD of the mean were classified as *low*, those scoring between –0.5 SD below and 0.5 SD above the mean were classified as *medium*, and those scoring 0.5 SD or above the mean were classified as *high*.

### 2.3 Procedure

The longitudinal ELISA project, initiated in 2017, is an ongoing study that has been continuously conducted up to the present day. This study has been approved by the Spanish Ministry of Economy and Competitiveness and by the University of Santiago de Compostela Bioethics Committee. Initially, 126 schools across urban, suburban, and rural areas in the Autonomous Community of Galicia (northwestern Spain) were contacted. Of these, 72 public (79.2%), charter (18.1%), and private (2.8%) schools agreed to participate. Subsequently, the families of the children were invited to participate, with ~25%–50% of families in each school agreeing to take part.

The child's primary caregiver (i.e., mother, father, or primary caregiver) completed a questionnaire at each collection point. Most respondents were mothers (87.3%). School teachers were responsible for supervising the distribution and collection of the questionnaires. Every part involved used a personal keycode to access and identify the questionnaires in order to ensure data confidentiality. Informed consent was obtained from the primary caregiver prior to each data collection. There was no financial compensation for participation.

To the extent possible, an attempt was made to standardize the administration of the questionnaires (from the order of presentation of the scales to the time and place where the questionnaires were administered) across the diverse range of schools included.^1^

### 2.4 Analyses

From the original sample of 1,352 children, discarding all the entries that miss any of the variables required is mandatory. Table 1 describes the final set of 20 variables featured in the analysis. Table 2 includes a definition of each variable extracted from the construction/validation of the instrument.

**Table 1:** Names and descriptive labels of the variables included in the Random Forest analyses.

**Variables**		**Name in database**	**Label**
Children's predictors (T1)	Temperament variables	TEMP_FOC.1	Attention focusing (TMCQ)
		TEMP_INHIB.1	Inhibitory control (TMCQ)
	Emotional variables	REG.1	Emotional regulation (ERC)
		LAB_NEG.1	Lability/Negativity (ERC)
		EMP_AF.1	Affective empathy (GEM)
		EMP_COG.1	Cognitive empathy (GEM)
	Psychopathic traits	GD.1	Grandiose-Deceitful (CPTI)
		INS.1	Impulsive-Need for stimulation (CPTI)
		CU.1	Callous-Unemotional traits (CPTI)
	Conduct problems	CP.1	Conduct problems (CPS)
Parenting predictors (T1)	Conflict	CONFLICT.1	Child-Parent conflict (CPRS-SF)
	Dysfunctional parenting practices	OVERREACT.1	Overreactivity (PS-8)
		LAXNESS.1	Laxness (PS-8)
	Warmth	WARMTH.1	Parental warmth (CRS)
	Stress	STRESS_PAR.1	Parenting stress (PSS)
Parents characteristics (T1)	Anxiety/depressive symptoms	PHQ_ANS.1	Parental anxiety (PHQ-4)
		PHQ_DEP.1	Parental depression (PHQ-4)
	Stress	STRESS_PERC.1	Parental perceived general stress (PSS-4)
	Perceived support	SUPPORT_E.1	Received emotional support (BRIEF-2)
		SUPPORT_I.1	Received instrumental support (BRIEF-2)
Outcomes (T2)	Children's conduct problems	CP.2	Conduct problems (CPS)

Suffix .1 indicates variables from the first wave of the study, suffix .2 indicates variables from the second wave.

TMCQ, Temperament in Middle Childhood Scale; ERC, Emotion Regulation Checklist; GEM, Griffith Empathy Measure; CPTI, Child Problematic Traits Inventory; CPS, Conduct Problems Scale; CPRS-SF, Child-Parent Relationship Scale-Short Form; PS-8, Parenting Scale Short Form; CRS, Child Rearing Scale; PSS, Parental Stress Scale; PHQ-4, Patient Health Questionnaire-4; PSS-4, Perceived Stress Scale-Short Form; BRIEF-2, BRIEF-2 Social Support Scale.

**Table 2:** Names and definitions of the variables included in the Random Forest analyses.

**Name in database**	**Definition**
TEMP_FOC.1	Tendency to maintain attentional focus upon task-related channels
TEMP_INHIB.1	The capacity to plan and to suppress inappropriate approach responses under instructions or in novel or uncertain situations
REG.1	Ability to monitor, evaluate and modify emotional reactions in an adaptive way
LAB_NEG.1	Lack of flexibility, anger dysregulation, and mood lability
EMP_AF.1	Affective response more appropriate to or congruent with someone else's situation than to one's own situation
EMP_COG.1	Ability to intellectually take the role of perspective of another person involving the ability to decode and label emotions and their situation clues
GD.1	Grandiose sense of self-worth, lying, and deceitfulness
INS.1	Need for stimulation/Sensation-seeking/Proneness to boredom, and Impulsivity
CU.1	Lack of remorse or guilt and callousness/lack of empathy
CP.1	Closely based on criteria of ODD and CD of the DSM-IV-TR
CONFLICT.1	Feelings of frustration, disagreement, or tension, indicating that communication or understanding between them is not flowing smoothly
OVERREACT.1	Emotionally intense responses to child's behavior
LAXNESS.1	Inconsistent and permissive parenting behaviors
WARMTH.1	Positive emotional tone in parent-child interactions
STRESS_PAR.1	Levels of stress experienced by parents
PHQ_ANS.1	Two core criteria for generalized anxiety disorder
PHQ_DEP.1	Two core criteria for depressive disorders
STRESS_PERC.1	Degree to which situations in one's life are appraised as stressful
SUPPORT_E.1	Receipt of empathy, concern, affection and encouragement
SUPPORT_I.1	Tangible practical support
CP.2	Closely based on criteria of ODD and CD of the DSM-IV-TR

All the variables have been rescaled from their original ranges to a standard scale (from 1 to 5) to ease the interpretation of the results. As this is a linear transformation, the original information of the rescaled variables remains unaffected.

Once the data set was filtered, the ML algorithm was trained using a 10-fold cross-validation strategy widely used in the literature (71). Following this method, the data set is partitioned into ten equal batches of samples distributed randomly. Then, a ten-step iteration process begins, where nine batches are used to train the model and the remaining one to test the algorithm. On each iteration, the batch is used for testing changes. Once the ten sets have been tested, the results can be assessed.

This study defined two different sets of metrics depending on the aspect being evaluated: regression or classification. For the regression, we chose the Pearson correlation coefficient (PCC) to assess its accuracy regarding the original values of the target variable:


(1) $$\begin{array}{l} \rho_{X,Y}=\frac{cov\left(X,Y\right)}{XY} \end{array}$$

We also observed the Mean Absolute Error (MAE) of the model to understand the magnitude of the errors in the predictions:


(2) $$\begin{array}{l} MAE=\frac{\sum_{i=1}^{n}|y_{i}-x_{i}|}{n} \end{array}$$

For the classification, once the samples were distributed into their corresponding groups according to their predicted score, we could observe the confusion matrix of the model, the global precision and recall, and, in particular, the recall for the group of high CP.


(3) $$\begin{array}{l} Precision=\frac{True\;Positives}{True\;Positives+False\;Positives} \end{array}$$

(4) $$\begin{array}{l} Recall=\frac{True\;Positives}{True\;Positives+False\;Negatives} \end{array}$$

A set of widely used ML models were tested to find the most accurate. Both performance and interpretability were taken into account.

We conducted these tests two times:

- **Case A**: using the previous CP score (CP.1) as a predictor.
- **Case B**: without using CP.1 as a predictor.

With these cases, we expect to conclude the real relevance of having previously measured CP for prediction of later CP scores.

## 3 Results

In this section, we present the statistical analysis results for the two scenarios proposed, with and without the use of the variable CP on T1. Only 1,095 out of the 1,352 children have the complete data in the required variables described in Table 1. Table 3 presents each variable's average and standard deviation with the remaining children on a scale from 1 to 5.

**Table 3:** Average score and standard deviation for studied variables.

**Variable**	**Average**	**SD**
TEMP_FOC.1	3.60	1.06
TEMP_INHIB.1	3.56	0.68
REG.1	4.35	0.52
LAB_NEG.1	1.86	0.48
EMP_AF.1	4.12	0.75
EMP_COG.1	4.28	0.83
GD.1	1.44	0.60
INS.1	2.30	0.79
CU.1	1.30	0.51
CP.1	1.43	0.44
CONFLICT.1	1.62	0.66
OVERREACT.1	2.44	0.61
LAXNESS.1	1.96	0.57
WARMTH.1	4.62	0.44
STRESS_PAR.1	1.73	0.68
PHQ_ANS.1	1.94	0.90
PHQ_DEP	1.79	0.83
STRESS_PERC.1	2.24	0.68
SUPPORT_E.1	4.50	0.80
SUPPORT_I.1	4.60	0.67
CP.2	1.36	0.41

Distributing the 1,095 children in the three groups previously mentioned, we have 249 high cases, 434 medium cases, and 412 low cases on CP.2. As this imbalance affects the representation of high cases, we opted for balancing the samples used for training. To establish a benchmark, we tested a set of models widely used in the literature for regression purposes such as: Linear Regressor (LR),^2^ Bayesian Ridge Regressor (BRR),^3^ Support Vector Regressor (SVR),^4^ Gradient Boosting Regressor (GBR),^5^ and a Multi-Layer Perceptron Regressor (MLPR) implemented using Tensorflow python library.^6^ All these models were trained using optimal hyperparameters.

We then found the best possible setting for each model using the GridSearchCV function from the Scikit-Learn python library.^7^ This function searches the optimal set of ML hyperparameters. Tables 4, 5 show the regression results for both cases.

**Table 4:** System results for Case A (sorted by PCC).

**Case A**	**PCC**	**MAE**	**Precision**	**Recall**
LR	0.787	0.19	0.663	0.63
BRR	0.784	0.192	0.664	0.628
**RF**	0.78	0.194	0.653	0.629
GBR	0.776	0.199	0.663	0.611
MLPR	0.709	0.224	0.574	0.549
SVR	0.295	0.275	0.385	0.397

**Table 5:** System results for Case B (sorted by PCC).

**Case A**	**PCC**	**MAE**	**Precision**	**Recall**
**RF**	0.699	0.224	0.643	0.548
LR	0.686	0.221	0.622	0.582
BRR	0.682	0.221	0.633	0.585
GBR	0.674	0.223	0.631	0.579
MLPR	0.639	0.237	0.568	0.515
SVR	0.286	0.275	0.385	0.398

With the results of the regressions, the system can draw the children into the three categories established for this study. On Tables 4, 5 we can observe the precision and recall of each model. Results for RF are similar or better than the rest of the models validating its selection for this study.

Although RF implies a greater computational cost and complexity compared to other models such as LR or BRR, its internal structure brings a singular advantage which makes it the optimal choice for studying the interactions between the variables studied. Table 6 contains the parameters that were tested for RF during the grid search phase.

**Table 6:** Random Forest hyperparameters.

**Parameter**	**Possible values**
n_estimators	25/50/100/250/500
max_features	None/“sqrt”
max_depth	4/5/6/7/8/9/10
min_samples_split	0.0001/0.001/0.01/0.1
min_samples_leaf	0.0001/0.001/0.01/0.1
criterion	“squared_error”/ “absolute_error”/ “friedman_mse”/“poisson”

For Case A, the best criterion for splitting the samples into different branches is the Poisson criterion, being the absolute error criterion for Case B. Both approaches share the rest of the optimal parameters, needing 250 estimators, with a maximum tree depth of 6 nodes, a sample split, and a leaf threshold of 0.001 (or 0.1% of the original samples). Both models were trained using their optimal configuration and a bootstrapping technique, which selects a random subsample of the data set for the training of each estimator, enhancing the accuracy of the regression.

**Figure 1:**
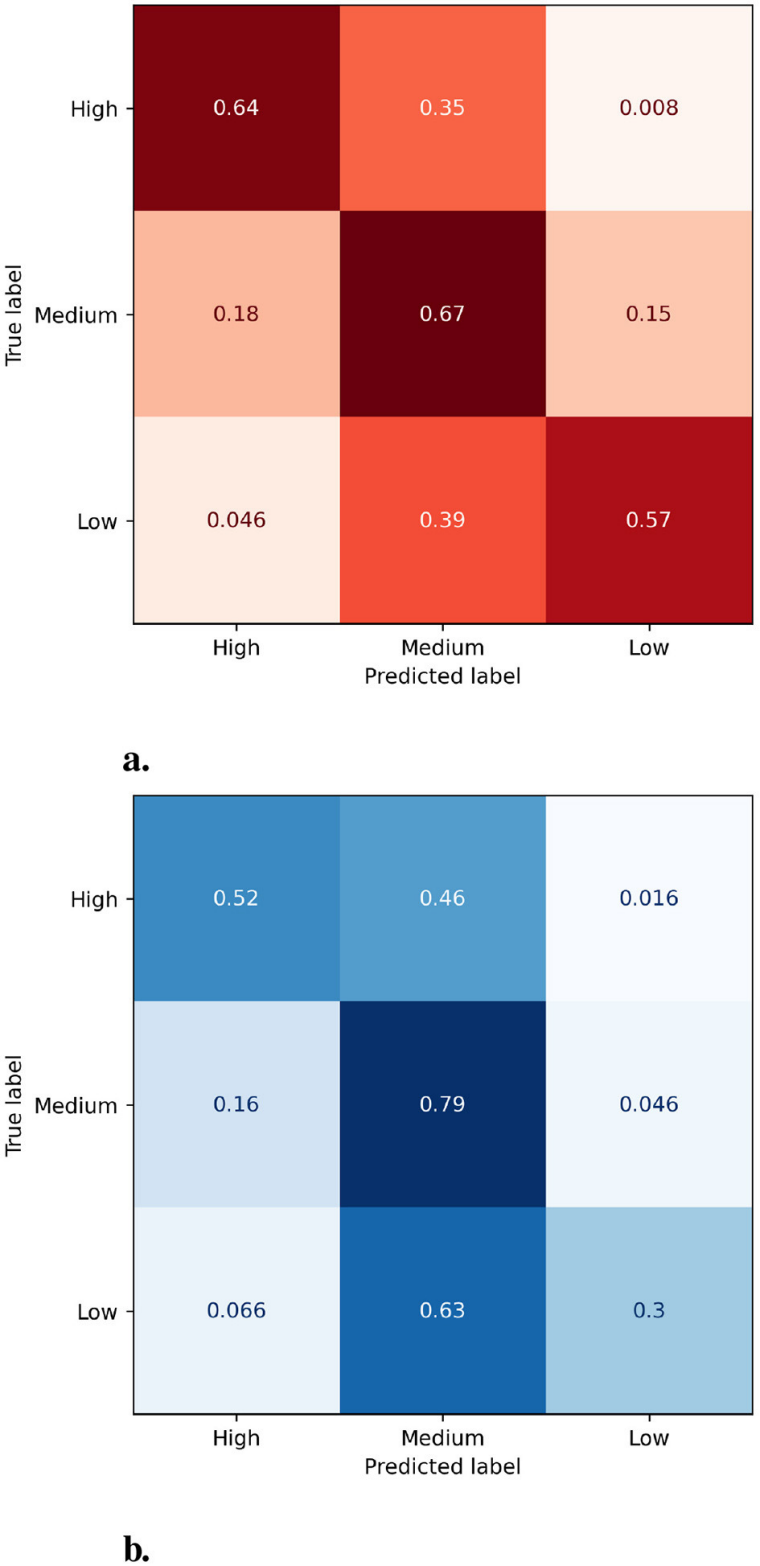
Heatmap representation of the confusion matrices for both cases. **(a)** Case A. **(b)** Case B.

Observing the PCC in both Tables 4, 5, we can see that the availability of records from previous years (CP.1) grants a better performance for the prediction of CP.2. In terms of MAE, Case A has an average error of 0.197 (48% of the SD of CP.2) vs. the 0.234 (57% of the SD of CP.2) from Case B. Figure 1 contains the confusion matrices for both scenarios with RF.

As we can see, the prediction for Case A offers a much better distribution of samples, with almost $\frac{2}{3}$ of each category correctly classified. This scenario has a global rate of success of 62.92%, 8% better than Case B.

**Figure 2:**
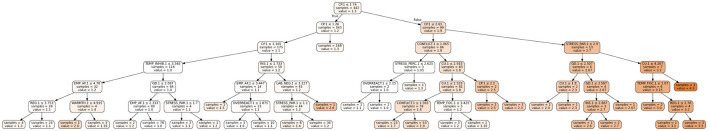
Graphical representation of a DT.

Once both models have been trained and tested, it is time to study their internal structures and observe the most frequent paths in the DTs that form the RF. Figure 2 includes an example of a visual representation of one of the 250 DTs conforming the RFs and all RF paths and thresholds are in Supplementary material.

Each node on the tree (except the leaf nodes) evaluates the value of one variable for all the samples that reach that node. The node sorts the samples depending on whether their magnitude for that particular variable is lower, equal, or higher regarding a threshold.

The field *Value* of each node represents the regression of the target variable (CP.2) for the set of samples at that particular node. The leaf nodes exist at the end of each branch and determine the final value of the regression for the samples that reach them.

This part of the analysis studies the presence or absence of the 19 (or 20 including CP.1) variables used for training the model, paying particular attention to their hierarchical distribution and combinations. When studying the structure of the trees, the level on which a variable appears may determine how relevant it is for the regression, as it implies its capability to classify larger groups of samples (e.g., the root node will be the one that can divide the complete set of samples most effectively).

A first approach, summarized in Tables 7, 8, observes the frequency of appearance of each variable on each level of the tree from 0 (root node) to 5, depending on whether the variable CP.1 was available or not.

**Table 7:** Depths for Case A.

**Variable**	**Depths**					
	**0**	**1**	**2**	**3**	**4**	**5**
CP.1	250	419	263	148	146	143
GD.1		34	88	139	169	242
TEMP_INHIB.1		19	130	178	173	256
CU.1		11	36	135	189	266
LAB_NEG.1		10	119	151	233	347
CONFLICT.1		4	89	154	239	296
WARMTH.1		2	36	81	173	231
REG.1		1	47	97	191	244
INS.1			56	115	186	277
STRESS_PAR.1			40	118	204	234
SUPPORT_E.1			20	64	137	166
TEMP_FOC.1			18	72	152	286
STRESS_PERC.1			13	88	177	213
EMP_COG.1			13	71	164	222
SUPPORT_I.1			7	54	103	170
PHQ_ANS.1			7	30	87	152
EMP_AF.1			4	51	139	205
PHQ_DEP			4	33	76	100
OVERREACT.1			3	50	134	198
LAXNESS.1			2	57	123	210

**Table 8:** Depths for Case B.

**Variable**	**Depths**					
	**0**	**1**	**2**	**3**	**4**	**5**
LAB_NEG.1	55	77	128	181	269	373
GD.1	45	72	96	134	214	312
CONFLICT.1	42	68	101	167	258	365
CU.1	26	49	107	138	170	265
INS.1	25	54	51	120	203	337
TEMP_INHIB.1	23	53	80	142	212	324
TEMP_FOC.1	9	32	55	101	216	359
STRESS_PAR.1	9	28	69	142	205	283
STRESS_PERC.1	7	5	30	70	151	316
REG.1	5	16	38	69	154	294
EMP_COG.1	2	11	29	70	134	249
WARMTH.1	1	6	42	81	161	280
PHQ_DEP	1	5	22	55	108	168
PHQ_ANS.1		7	16	55	112	213
EMP_AF.1		6	27	63	134	206
LAXNESS.1		4	33	108	197	299
SUPPORT_I.1		3	22	57	110	209
SUPPORT_E.1		2	24	56	121	204
OVERREACT.1		2	22	70	174	286

As we can observe, CP.1 is the most relevant variable to predict CP.2, being the root node at every tree in the RF. It is also the predominant variable for levels 1 and 2. This means that, by establishing multiple thresholds for CP.1, the algorithm can execute a coarse classification of samples.

We should expect that the relevant variables in Case B would be the first ones to appear in Case A, too. As it is true for GD.1, we can see that variables like LAB_NEG.1 or CONFLICT.1, which are two of the most frequent root variables for Case B, are not that relevant compared to others like TEMP_INHIB.1 or CU.1 in Case A. These first results can offer powerful insights on which variables can be targeted when studying CP.

When studying the structure of the trees, it is important to observe not only the position of the variables but also how they combine to form paths in the RF. These combinations can highlight smaller subsets of variables reliable for classification. Shorter paths with strong performances may simplify future longitudinal studies by reducing the necessary number of variables needed to provide accurate predictions on CP.

Table 9 shows a concise tabular representation of the paths contained in the trees described above. As stated earlier, all the paths studied from now on lead to high values of CP.2.

**Table 9:** Example of the tabular representation of a path.

**Path**	**Node 0**	**Node 1**	**Node 2**	**Node 3**	**Node 4**	**Node 5**	**Leaf samples**	**Precision**
17	temp_inhib.1	GD.1	LAB_NEG.1	CONFLICT.1	temp_inhib.1	EMP_AF.1	13	100%
	3.34	2.07	2.17	3.57	3.07	1.67		

Variables in capital letters mean values above the threshold; lowercase means equal or below.

A path can have a maximum of six nodes. Each row contains the variables evaluated in each node and the thresholds established to sort the samples left (variable equal or lower than the threshold) or right (variable higher than the threshold) in the trees. The thresholds are averaged if there are more than one occurrence of a particular path.

If the variable's name is presented in capital letters, it means that the samples present a higher value than the threshold for that particular variable. Otherwise (with a lower or equal score), the variable is presented in lowercase letters (i.e., Table 9 shows a path leading to high CP.2 where temp_inhib.1 was lower than 3.07; GD.1 was higher than 2.07; LAB_NEG.1 was higher than 2.17; CONFLICT.1 was higher than 3.57 and EMP_AF.1 was higher than 1.67, all of them scored over five points).

Additionally, we present the average number of samples that met all the conditions of a particular path and the precision that it accomplishes for high CP.2 prediction.

We opted to study the common roots to obtain representative subsets of variables instead of full paths. We define these roots as a set of the first N nodes of a path. This allows to merge similar paths into prototypical groups of variables.

We chose the size N that gave us roots with at least ten occurrences each in order to ensure meaningful results. For Case A, our roots contain four nodes, while for Case B, only three nodes are necessary. Tables 10, 11 present the top five most frequent roots for both Case A and Case B.

**Table 10:** Five most frequent roots for Case A.

**Node 0**	**Node 1**	**Node 2**	**Node 3**	**Avg. length**	**Count**	**Avg. leaf samples**	**Precision**
cp.1	cp.1	CP.1	temp_inhib.1	5.93	103	2.36	51.03%
1.64	1.24	1.05	3.37				
cp.1	cp.1	temp_inhib.1	stress_perc.1	5.60	56	3.48	40.51%
1.62	1.22	3.46	1.41				
CP.1	cp.1	cp.1	CONFLICT.1	5.93	43	29.05	50.44%
1.51	2.71	1.92	1.95				
CP.1	CP.1	gd.1	reg.1	5.88	42	6.79	84.21%
1.44	2.16	3.23	3.71				
CP.1	CP.1	gd.1	REG.1	5.84	34	14.71	75.40%
1.49	2.11	3.28	3.68				

Variables in capital letters mean values above the threshold; lowercase means equal or below.

**Table 11:** Five most frequent roots for Case B.

**Node 0**	**Node 1**	**Node 2**	**Avg. length**	**Count**	**Avg. leaf samples**	**Precision**
INS.1	gd.1	CONFLICT.1	5.89	18	14.78	63.53%
2.49	3.19	2.30				
gd.1	LAB_NEG.1	CU.1	5.71	17	6.47	57.27%
1.78	1.93	1.69				
GD.1	LAB_NEG.1	lab_neg.1	5.94	16	11.63	76.88%
1.76	2.35	3.48				
CONFLICT.1	gd.1	CU.1	5.73	15	19.53	64.51%
1.89	3.54	1.47				
INS.1	cu.1	CONFLICT.1	5.93	15	20.73	56.91%
2.48	2.19	1.98				

Variables in capital letters mean values above the threshold; lowercase means equal or below.

The column **Avg. Length** presents the average length of the paths that start with the roots presented in the tables. The column **Count** presents the number of paths that contain a particular root in all the 250 DTs included in the model. The column **Avg. Leaf samples** describes the average number of samples sorted in the leaves of the paths that start with said roots. Finally, the column **Precision** is the average precision of all the paths starting with the same root.

Ideally, relevant roots should be highly frequent, with high precision and, as a lesser requirement, leading to shorter paths. That would mean that only N variables would be needed to predict CP and thus would ease the data gathering process.

As shown in Table 10, the most frequent roots in Case A combine the previous measure of CP with several individual (i.e., inhibitory control, GD, emotional regulation) and family (parents' perceived stress, parent-child conflict) variables. In Case B (Table 11), psychopathic traits (INS, GD, CU) tend to appear combined with different levels of other individual variables (lability/negativity) and also with parent-child conflict.

However, we wanted to know the best roots for each case. We defined an *ad-hoc* metric for assessing the quality of the roots. The metric is defined as follows:

1. The root must have a **Precision** equal to or higher than the global precision of the model.
2. The root **Count** must have at least 10 samples.
3. The value is obtained using the harmonic mean of the normalized values of the **Precision** and the **Count** of each row as follows:

(5) $$\begin{array}{l} P=\frac{precision}{max\left(Precision\right)} \end{array}$$

(6) $$\begin{array}{l} C=\frac{count}{max\left(Count\right)} \end{array}$$

(7) $$\begin{array}{l} M=\frac{2*P*C}{P+C} \end{array}$$

Thus, the best roots are those that most frequently appear and, at the same time, have a higher success rate in the classification task. Tables 12, 13 present the five best roots for Case A and Case B, respectively.

**Table 12:** Five best roots for Case A.

**Node 0**	**Node 1**	**Node 2**	**Node 3**	**Avg. length**	**Count**	**Avg. leaf samples**	**Precision**	**M**
CP.1	CP.1	gd.1	reg.1	5.88	42	6.79	84.21%	0.91
1.44	2.16	3.23	3.71					
CP.1	CP.1	gd.1	REG.1	5.84	34	14.71	75.40%	0.78
1.49	2.11	3.28	3.68					
CP.1	cp.1	CP.1	CU.1	5.79	34	16.82	72.55%	0.77
1.49	2.55	1.83	1.87					
CP.1	cp.1	CP.1	cu.1	5.97	33	33.18	65.48%	0.71
1.50	2.56	1.86	2.13					
CP.1	CP.1	cp.1	gd.1	5.86	29	19.14	72.07%	0.71
1.41	2.22	3.13	3.37					

Variables in capital letters mean values above the threshold; lowercase means equal or below.

**Table 13:** Five best roots for Case B.

**Node 0**	**Node 1**	**Node 2**	**Avg. length**	**Count**	**Avg. leaf samples**	**Precision**	**M**
LAB_NEG.1	gd.1	CU.1	6.00	15	7.60	82.46%	0.85
2.28	3.56	1.80					
GD.1	LAB_NEG.1	lab_neg.1	5.94	16	11.63	76.88%	0.85
1.76	2.35	3.48					
LAB_NEG.1	reg.1	LAB_NEG.1	5.85	13	4.38	85.96%	0.80
2.19	3.42	2.61					
INS.1	gd.1	CONFLICT.1	5.89	18	14.78	63.53%	0.80
2.49	3.19	2.30					
GD.1	CONFLICT.1	STRESS_PAR.1	5.67	12	2.33	92.86%	0.79
1.73	3.12	2.80					

Variables in capital letters mean values above the threshold; lowercase means equal or below.

The best roots when previous CP is included (Case A, Table 10) replicated, to a great extent, the patterns of the most frequent roots: different levels of CP cluster together with inhibitory control, GD, and emotional regulation. CP also appears combined to high CU and with parents' perceived stress. For Case B (Table 11), psychopathic traits again appear in combination with lability/negativity and with family variables (parent-child conflict and parenting stress). The highest precision (92.86%) is achieved by high levels of GD in association with high conflict and parenting stress. Additionally, high levels of lability/negativity and low emotional regulation define one of the most accurate paths (86.96%) for this case.

## 4 Discussion

This study explored the utility of RF models for predicting high CP in children based on individual and family factors measured in a longitudinal design. Many decades of research have suggested a plethora of variables potentially involved in the development of CP, with studies arising from a variety of theoretical and empirical perspectives. In this research, the use of ML (and, particularly, RF) has allowed us to go beyond previous approaches by using a more flexible, data-driven approach that can process a high volume of data with fewer statistical assumptions.

The specific focus of this study was to identify the most relevant individual and family predictors and to capture interactions among them. In the field of individual factors, we included both dispositional traits (e.g., temperament, psychopathic traits) and socioemotional competence (e.g., emotional regulation, empathy); as for family factors, we considered not only variables of family functioning (e.g., conflict, parenting practices) but also parents' personal conditions (e.g., stress, depression) that have been previously proposed as sources of behavioral disturbances in children [e.g., (35, 37)], thus covering a wide array of factors distilled from more traditional studies on CP.

This study evaluated two different models: Case A included the previous measure of CP as a predictor, while Case B removed the previous measure of CP, to determine how individual and family factors could be “purer” predictors without prior data on CP. Results showed that both overall prediction and specific prediction of high CP performed better in Case A, where previous CP was incorporated. This expected result reflects a well-known behavioral science principle: past behavior is the best predictor of future acts [e.g., (72)]. Within the specific field of CP in children, this is also a commonly found pattern [e.g., (73, 74)], which reflects the relative stability of CP over time (75), and, in practical terms, it shows that information about past problems is a strong indicator of risk for later CP. Nevertheless, the results also suggest that, beyond the information provided by the previous measure of CP, additional factors emerge as relevant for the classification of children according to their probability of future CP.

### 4.1 Identification of the most relevant predictors

Among the variables used for model training, some stand out for predicting future CP, considering their positions in the tree structures. A first noteworthy result is that individual factors play a more decisive role than family factors, regardless of whether previous CP are considered. This is consistent with findings from other studies that compared factors from different domains to predict mental health outcomes in youth (38).

This pattern of findings can also be considered in the light of broad theoretical frameworks in behavioral sciences like Bronfenbrenner's (76, 77) Bioecological Model. According to this model, human development results from several intertwining layers of influence, including the individual and progressively larger systems (e.g., family, community, cultural values, etc.); despite the powerful influence of the family contexts, individual characteristics are more directly related to behavior, and, ultimately, filter the experiences and interactions with the other layers.

Within the individual factors, our results highlight the role of temperament and psychopathic traits in the pathways to children's CP. Particularly, the results reinforce the role of effortful control (attentional focusing and, especially, inhibitory control), in alignment with other studies (78), that highlight self-regulatory dispositions as predictors of behavioral disturbances. Our results also point to emotional reactivity (lability/negativity) as a significant contributor to the prediction of CP. This result is consistent with a strong line of research on the role of negative affect (79), emotional instability (80), and irritability (81) in the configuration of the children's most common CP. Thus, RF models endorsed both the tendency to display intense emotionality and the difficulties for self-control as key factors in the prediction of CP.

Our results also bolster the role of the so-called psychopathic traits (82) for the identification of children at risk of high CP. The significance of psychopathic traits remains evident, regardless of whether prior CP are included as a predictor. Results show that CU, often considered the core of psychopathic personality (83), is located within the highest positions in the decision trees. Thus, emotional coldness, lack of guilt, and insensitivity to other needs, which define the concept of CU, are corroborated as powerful aspects for the detection of risk for high CP. However, results indicate that other psychopathic variables are also important. GD ranks even higher than CU both in Case A and Case B; it seems that interpersonal components of the psychopathic domain (e.g., arrogance, manipulativeness, disposition to cheat or lie for the own benefit) play a standing role in the prediction of CP. Also, at least in Case B, the behavioral facets of the psychopathic traits (INS), encompassing impulsivity, irresponsibility, and need for stimulation, are part of the most influential predictors. Therefore, results bring support for a burgeoning research line claiming that, besides CU traits, interpersonal and behavioral psychopathic traits should be considered for a more precise account and prognosis of high CP (21, 22).

In the family realm, results underscore the role of conflict in the relations between parents and children. This result is in keeping with previous research showing how developmental outcomes are strongly influenced by the family atmosphere, family strain, and difficult interactions between parents and children [e.g., (84, 85)]. In our study, although some other family variables (e.g., parenting stress) seem decisive in the highest nodes, conflict stands out among the other family factors in both Case A and B. The power of this dimension may respond to the fact that conflictive relations can inherently reflect other relevant family aspects, like ineffective parenting and a compromised emotional connection among the family members (86, 87), thus being an efficient indicator of broader dysfunction in the family setting. Therefore, we found that the most important variables align with findings from previous literature on CP. The RF models underscore a specific set of predictors, both at the individual and family levels, that have been emphasized in psychological research and theory. This convergence between computational findings and existing research is noteworthy, as it not only validates the identified predictors, but also reinforces some previous advances in developmental psychopathology. At the same time, the RF models offer valuable insights for further exploration. Future research should keep expanding beyond the central focus on CU traits when studying psychopathic tendencies at early ages, and place greater emphasis on the core role of parent-child conflict among the various family factors typically considered in the development of CP.

### 4.2 Relevant combinations of predictive factors

In addition to studying the most relevant variables for prediction of CP, we also sought to identify clusters of variables that tend to act together, in an attempt to capture the interaction patterns that emerge from the RF analyses. Both the most frequent and the most successful combinations were analyzed, and similar combinations were identified in both approaches. Some sets of variables bring together different kinds of individual factors. Specifically, psychopathic and temperamental predictors tend to appear together in a relatively high number of samples and are also part of the most accurate paths. For example, GD and lability/negativity align together frequently and successfully. Some other times, several psychopathic traits are combined with lability/negativity.

Also, the tandem of GD and social competence (emotional regulation) can be identified, particularly when CP are also included as predictors. These results suggest the need for further studying the interactions between psychopathic and affective/self-regulatory traits [see, for example, (88, 89)] for a better depiction of the psychological mechanisms underlying high CP.

The analysis of predictor combinations also points to some individual and family factors working together in the classification task. For example, inhibitory control and parents' stress show up as a common and accurate cluster, a pattern of results that reflects the relevance of studying the interactions between temperament and family, as several previous research lines have suggested (27, 90). Also, our results show that psychopathic traits and family dimensions repeatedly appear as sets of intertwined variables. Specifically, interactions identified by the RF models highlight the predictive power of high levels of parent-child conflict when combined with CU or INS. Additionally, the coexistence of high GD, parent-child conflict, and stress in the parental role defines a particularly risky setting for the development of CP. As previous research has shown, psychopathic factors could trigger family dysfunction (91), or, conversely, the family dynamics could affect the development of psychopathic traits (92); psychopathic traits could also moderate the impact of family factors on developmental outcomes (93). Our results suggest that the dynamics of psychopathic traits and family relations (particularly, the strained relations between parents and children) should be a prioritized research field, so that the predictive and etiological processes underlying CP can be fully understood.

### 4.3 Theoretical and practical implications

Results from RF can provide valuable orientations for theory building and refinement in the field of early CP. The findings on individual dispositions (temperament and psychopathic traits) suggest that models on CP should account not only for these factors but also for their joint effect and their interaction with family variables. Emotional reactivity and self-regulatory skills are highlighted as two main axes of temperamental influence; both axes have already been present in classical models on “difficult temperament” (94) and in theoretical models specifically designed for antisocial behavior [e.g., (95)]. Results from RF corroborate the impact of such dimensions and support their utility in the design of broader models of CP. Also, psychopathic traits should occupy a significant space in theories on stability, chronicity, and severity of CP; within this framework, not only does affective insensitivity (CU) seem relevant, but behavioral traits (INS) and, especially, the interpersonal aspects (GD; manipulativeness, narcissism, dishonest charm) should also be considered (21, 82). These dispositions should be addressed in interaction with the family function; particularly, according to our results, and in line with coercion theory (66) conflictive interactions between parents and children, possibly within a psychosocially stressed family, may be a key factor for etiological models of children's CP.

Additionally, insights from RF provide support to the developmental heterogeneity of CP (96). As shown by our results, CP can be predicted on the basis of different combinations of factors; in some of the trees, individual factors (e.g., GD and lability/negativity) seem to be dominant, while in others, both individual and family factors play a pivotal role in prediction roots (e.g., family conflict, GD and CU). In this sense, RF models can be a useful way to explore the variety and complexity of pathways leading to CP, resonating with the principles of equifinality and multifinality proposed by developmental psychopathology (97).

On a practical level, our results can help in the early identification of at-risk children by recognizing the main predictive variables, the more powerful clusters of factors, the thresholds and the itineraries for making classificatory decisions. Beyond the previous levels of CP, individual and family factors can aid in distinguishing children who may present future behavioral difficulties. This can guide social and health policies so that targeted interventions can be designed to curb the problematic pathways. Additionally, our results yield meaningful information for guiding prevention programs by highlighting major domains for intervention like interpersonal sensitivity, coping with frustration and negative emotions, executive functioning skills, and parent-child relational patterns. Some interventions targeting these factors have demonstrated their efficacy for prevention of CP, thereby reinforcing the relevance of these predictors as key contributors to the development of CP. For instance, some interventions have built on the child's individual characteristics to promote cognitive flexibility and constructive coping strategies [e.g., Problem-Solving Skills Training (PSST)] (98). Other interventions, like the Triple P-Positive Parenting Program (99) have focused on family dysfunction, and have shown significant success in addressing CP. Moreover, the combined influence of individual and familial predictors in CP prediction further supports the need for a multicomponent approach that targets a broader range of risk factors. Such an approach may be particularly beneficial in CP treatment, as demonstrated by programs like Incredible Years (100), which have shown strong short- and long-term efficacy. Based on our results, further research should advance in the design of individual- and family-oriented programs that consider the diversity of needs of CP children. In particular, modular programs [e.g., (101)], matching the intervention components to the strengths and difficulties of each child, would show promise for dealing with the heterogeneity of pathways in children's CP.

### 4.4 Limitations and suggestions for further research

The results of this study should be viewed in light of some limitations. First, despite the longitudinal nature of the design, which enables prospective predictions not attainable by cross-sectional research, the follow-up (1 year) was relatively short. We analyzed data from children across the elementary school years, a critical period for the development of the most stable patterns of CP; however, prediction through a longer time frame, including the preschool years, would be particularly useful for identifying the earliest predictors and informing preventive practices in early childhood.

Second, while the ELISA sample was relatively large and reasonably heterogeneous, it was community-based and drawn from a specific sociocultural setting, which may restrict full generalizability to other samples and settings. Additionally, in studies like ours, the likelihood of involving children with very high CP is moderate unless there is an oversampling of participants exhibiting significant difficulties at baseline. Therefore, caution is advised, as the results might not be fully applicable to other groups. Further studies in diverse populations and social backgrounds are needed to evaluate the robustness and generalizability of our findings. Such research will help clarify which predictors of CP are consistent across different contexts and provide a more refined perspective on CP prediction. It is also worth noting that in this study, we excluded children who missed any data collection points. Additionally, previous studies associated with the ELISA project (32, 102), as well as existing literature (103), have shown that children with incomplete assessments typically have a lower socioeconomic status (SES) compared to those with complete data. This may introduce bias and limit the generalizability of the findings to children from lower SES backgrounds. Therefore, it is recommended that future studies place a stronger emphasis on retaining participants from disadvantaged backgrounds, as they are more likely to drop out of community-based longitudinal studies.

Third, our selection of family variables prioritized malleable factors, which are particularly useful for informing the design of preventive interventions. Consequently, certain variables, such as socioeconomic background, parental education level, and the presence of serious mental disorders in parents, were not included in the model. Research has shown that these factors can significantly affect developmental outcomes, including CP, often indirectly [e.g., (104)]. Incorporating such variables into future models could help further elucidate their role within a more comprehensive framework of risk factors.

Future research should address these limitations. As suggested above, applying RF to long-term longitudinal data will enhance the opportunity for predictions across extended periods of the lifespan. Additionally, examining predictors measured at different ages (e.g., preschool and primary school) could help identify which factors are more significant at different developmental stages and depict how the pathways to CP unfold through time. This approach would enhance prediction and theory building and improve prevention by highlighting critical ages for timely intervention and indicating which factors should be addressed at different developmental times.

Future investigations should also refine the RF models by incorporating a wider range of potential predictors. While individual and family factors are recognized as fundamental domains underlying CP [e.g., (105)], other variables from the community, school and peer contexts could boost the predictive power of the RF and provide new insights on the interactions that drive CP.

Also, RF models in this field can be fine-tuned by considering how the pathways to CP may be conditioned by gender. Higher prevalences of CP in boys are commonly found (106), although when CP are developed in girls, the behavioral difficulties may be particularly detrimental [i.e., the “gender paradox”; (107)]. Nevertheless, the differential factors involved in the development of CP in boys and girls are poorly understood (108). Longitudinal studies with RF can be a useful tool to unravel the distinctive routes to CP across genders.

### 4.5 Concluding remarks

This study illustrates how explainable ML can integrate predictive accuracy with psychological understanding, elucidating not only the key variables, but also the ways in which they interact across multiple levels of influence to contribute to maladaptative developmental trajectories. Data-driven models based on RF algorithms represent a promising approach to address the complexities of predicting and explaining CP. Our results emphasize the influence of individual and family factors and demonstrate how these elements are intricately combined to shape heterogeneous pathways. Moreover, findings from this longitudinal study can inform more effective screening processes and tailored interventions; they also highlight the value of bridging psychological insights and advanced computational methods to better address one of the most prevalent and impairing challenges in children's mental health.

## Data Availability

The raw data supporting the conclusions of this article will be made available by the authors, without undue reservation.

## Footnotes

^1^See http://www.personalitydevelopmentcollaborative.org/project-page-elisa/ for additional details.

^2^Available at: https://scikit-learn.org/stable/modules/generated/sklearn.linear_model.LinearRegression.html, October 2024.

^3^Available at: https://scikit-learn.org/stable/modules/generated/sklearn.linear_model.BayesianRidge.html, October 2024.

^4^Available at: https://scikit-learn.org/stable/modules/generated/sklearn.svm.SVR.html, October 2024.

^5^Available at: https://scikit-learn.org/stable/modules/generated/sklearn.ensemble.GradientBoostingRegressor.html, October 2024.

^6^Available at: https://www.tensorflow.org/api_docs/python/tf/keras, October 2024.

^7^Available at: https://scikit-learn.org/stable/modules/generated/sklearn.model_selection.GridSearchCV.html, October 2024.
